# Supplementary material for: Genomic sequencing of SARS-CoV-2 in Rwanda reveals the importance of incoming travelers on lineage diversity
Source: Nat Commun. 2021 Sep 29;12:5705. doi: 10.1038/s41467-021-25985-7 (PMC8481346; doi:10.1038/s41467-021-25985-7)
Supplement: Supplementary file 1 — Supplementary Information [file 41467_2021_25985_MOESM1_ESM.pdf]

Genomic sequencing of SARS-CoV-2 in Rwanda reveals the importance of incoming travelers on lineage diversity

Emergence and importance of A.23.1 and B.1.380 in Rwanda

We here discuss the impact that the lineages discussed in the main text have had on the local transmission dynamics in Rwanda. We note that this discussion is highly dependent on the sampling efforts in Rwanda and its neighbouring countries and want to caution against overinterpretation and consider these findings against the ratio of available genomes versus overall case counts. To compare the epidemic situation in Rwanda against that in its neighbouring countries, we focus on the two countries that our results point to as being most related - in terms of viral exchanges - to Rwanda, i.e. Uganda and Kenya.

To this end, we have created custom frequency plots for the purpose of assessing the impact of the two main lineages we have focused on, i.e. A.23.1 and B.1.380. We retrieved all available SARS-CoV-2 genomes and lineage annotations from GISAID across the time frame of reference for our study, and calculated the proportion of sequences collected in a given day for each lineage. The resulting time series were smoothed using a second-order Savitzky-Golay filter on 61-time windows (Savitzky and Golay, 1964), as implemented in the SciPy Python library (Virtanen et al., 2020). These frequency plots are shown in Supplementary Figures S6 (Rwanda), S7 (Uganda) and S8 (Kenya). Note that it is highly important to jointly consider both figure panels for a correct interpretation.

Our results point to B.1.380 having become the dominant lineage and being responsible for the total case count in Rwanda since its emergence in the second half of 2020 and until the emergence of A.23.1 (see below). This pattern is not encountered in neither Uganda nor Kenya, where B.1.380 seems to be quite irrelevant. Further, our results show a similar pattern in the

emergence and increase in infections of lineage A.23.1 in Rwanda as in Uganda, albeit with a delay of roughly six weeks. As more and more samples were being sequenced as of the second half of December 2020, most of the infections in Rwanda could be attributed to A.23.1, with an increasing proportion of B.1.351 since the start of 2021. It hence seems fair to assume that the epidemic situation in Rwanda and Uganda are linked (to some extent), and that A.23.1 was the driving factor behind the increase in total case counts in both countries. This is clearly not the case in Kenya, where the epidemic situation is markedly different, and the proportion of A.23.1 infections was only on the rise at the end of our study period but had not (yet) attained dominance.

Our phylogeographic analysis (see Figure 5 in the main text) estimates the origin of A.23.1 to have been in Uganda, corresponding with the observation that such genomes have been detected earlier in Uganda compared to Rwanda. The key difference (between Rwanda and Uganda) lies with the lineages being present before the increase in infections with A.23.1. Rwanda was heavily impacted by infections with lineage B.1.380 - a phenomenon not shared by Uganda and Kenya, who saw virtually no such infections - whereas Uganda saw mostly infections with a wide range of lineages - but mostly B.1 and A.23 - and Kenya with lineages B.1 and B.1.1. Our phylogeographic analysis (see Figure 6 in the main text) estimates the origin of B.1.380 to have been in Kenya, with such genomes being reported during a relatively short time before those from Rwanda and during the first part of the B.1.380 epidemic in Rwanda. This possibly points to a short B.1.380 epidemic in Kenya, although this is difficult to conclude due to the low total genome count. It is thus clear that each of these countries reacted differently - in terms of the relative proportion of infections - to the appearance of these lineages, possibly related to the lineages already circulating in those countries at that time.

Quantifying the relative contribution of introductions and local transmission is challenging in the case of limited genomic surveillance and we here refrain from comparing the estimated number of Markov jumps in our study to any size assessment of local transmission clusters in Rwanda. We have discussed in the main text that the BaTS analyses we performed indicated that local transmission played a more important role in driving the Rwandan epidemic compared to introductions, and this for both A.23.1 and B.1.380 lineages. This can also be seen in our phylogeographic analyses (Figures 5 and 6 in the main text) of these lineages, which are in stark contrast with studies that have reported the importance of introduction events, without explicitly quantifying the relative importance versus local transmission (e.g. Dellicour et al., 2021; Figure 1). Additionally, and as can be seen from Supplementary Figures S6-S8, estimates are easily biased as a result of a few single genomes, which demands caution against overinterpreting transmission cluster sizes and the interplay between introductions (of which many occurred via land travel and through international flights; see Supplementary Figure S2) and local transmission. Previous studies on the epidemic in Uganda (Bugembe et al., 2021) and Kenya (Githinji et al., 2020) have also refrained from drawing strong conclusions, which requires a highly representative genome sequencing effort not only in the country under study but also the main countries with which viral exchange takes place. An example can be found in a large-scale analysis in the United Kingdom (Du Plessis et al., 2021), where more quantitative analyses can in fact be used for this purpose.

A note on exploiting individual travel histories through phylogeographic reconstruction

The importance of using metadata associated with genomes in the form of individual travel histories has been shown in the work of Lemey et al. (2020) by an explicit (and time-consuming)

comparison of performing Bayesian phylogeographic inference with and without these travel data, on two different SARS-CoV-2 data sets. Rather than repeating this type of comparison, we here compare these travel records to the estimated Markov jumps between countries, for both key lineages analysed in our manuscript. Supplementary Figures S9 and S10 show this comparison for lineages A.23.1 and B.1.380, respectively. These figures show that the travel history-aware phylogeographic reconstruction (Lemey et al., 2020) does not merely report the individual travel histories but shows clear differences - in both directions - between the estimated number of Markov jumps (with Bayes factor >3) and the number of recorded individual travel histories between pairs of countries. In general, it is to be expected that the total number of transitions (Markov jumps) between countries along the entire estimated phylogeny will be higher than the recorded travel cases, as typically only a fraction of the genomic samples are accompanied by travel information. However, and as shown in Supplementary Figure S9, in some cases the inferred number of transitions between countries will be lower than the number of travel cases between those countries, for example when samples that correspond to known travel cases cluster together (e.g. in a hypothetical case of an infected family of four crossing a country border, which will result in four individual travel histories but only in a single Markov jump, on the assumption that they have a single / identical source of infection).

Assessing sampling bias in continuous phylogeographic analysis

The continuous phylogeographic analysis discussed in the main text, and shown in Supplementary Figure S11, focuses on a within-Rwanda analysis of viral spread. The sequencing effort described in this study yielded genomes that for a large part originated from Kigali, as described in the main text. While the precise sampling location within the country is not directly

relevant for discrete phylogeographic analysis, as performed in the main text, recent work does indicate that continuous phylogeographic reconstructions can be affected by this type of sampling bias, i.e. the lack of sampling from certain areas (Kalkauskas et al., 2021). However, given that international travel has been shown to be an important predictor for the spread of SARS-CoV-2 (Lemey et al., 2020) and that population density is highest in the province of Kigali, it seems reasonable to focus a fair share of genome sequencing efforts to this particular region of the country, i.e. in the region that also harbours one its main points of entry. As a result, the sampling bias will likely not lead to a shift in inferred location of origin as would be the case when sampling from only the eastern or western-side of the country for example (Kalkauskas et al., 2021).

When it comes to reconstructing the dispersal within Rwanda, the relatively higher sampling effort in Kigali indeed impacts the continuous phylogeographic reconstruction in the sense that such an analysis likely fails to highlight local circulation of lineages outside Kigali as a consequence of under-sampling in those regions. While the heterogeneous sampling effort (or sampling bias) indeed prevents us from interpreting this reconstruction as a realistic overview of the overall dispersal history of SARS-CoV-2 lineages in Rwanda, it still aims to infer the dispersal history of those lineages that were sampled in our study. Although a particular sampling will always affect the reconstructed dispersal history of viral lineages, continuous phylogeographic inference will still provide movement data that can inform on the dispersal dynamics of the virus (Dellicour et al., 2019). However, it stands to reason that investigating the local dispersal dynamic of viral lineages outside Kigali would certainly add much interesting information to this type of analysis.

In order to assess the impact of the sampling bias in our data set, we provide a sensitivity test by redoing our continuous phylogeographic analysis on ten subsampled data sets with reduced heterogeneous sampling, by downsampling the more intensively sampled areas. To this end, we have randomly selected a maximum of two sequences per administrative "sector" area in each of ten replicates continuous phylogeographic analyses. We show the results in Supplementary Figure S12, which illustrates that the phylogeographic reconstructions in these ten replicates are coherent with the phylogeographic dispersal pattern we inferred from the original data set.

Supplementary Tables

Supplementary Table S1. Non-pharmaceutical interventions by announcement date in Rwanda with key changes described. Sources are official government communiques, provided beneath the table.

Date	Details	Source
2020-03-22	· Unnecessary movements and visits outside the home are not permitted except for essential services such as healthcare, food shopping, or banking, and for the personnel performing such services<br>· Hand hygiene, social distancing, mask wearing<br>· Schools and places of worship are closed<br>· All borders are closed, except for goods and cargo, as well as returning Rwandan citizens and legal residents, who will be subject to mandatory 14-day quarantine at designated locations<br>· Travel between cities and districts of the country is not permitted, except for medical reasons or essential services; transport of food and essential goods will continue<br>· Shops and markets will remain closed, except those selling food, medicine, hygiene and cleaning products, fuel, and other essential items<br>· All bars are closed	1

2020-05-01	· Movements are prohibited from 8pm to 5am except with permission<br>· Mass screening and testing<br>· Public and private businesses will resume with essential staff while other employees continue working from home<br>· Markets will open for essential vendors not exceeding 50% of registered traders<br>· Manufacturing and construction sectors will open with essential workers<br>· Hotels and restaurants will operate but close at 7pm<br>· Individual sporting activity in open spaces is permitted, however sports facilities shall remain closed<br>· Public and private transport will resume within the same province<br>· Funeral gatherings should not exceed 30 people	2
2020-07-30	· Kigali International airport (KIA) reopen and tourism to resume<br>· Hotels resume activities<br>· Land borders will remain closed, except for goods and cargo	3
2020-08-14	· Passengers arriving at KIA must present a negative COVID-19 PCR test taken within 120 hours prior to departure<br>· Places of worship shall operate upon compliance with COVID-19 preventive measures	4
2020-08-27	· Authorized public gatherings, including conferences and weddings will resume, in adherence with health guidelines, including negative COVID-19 test and participants not exceeding 30% of the venue's capacity	5

2020-09-11	· School will resume with a gradual opening in the coming weeks<br>· Public transport between Kigali and other provinces will resume<br>· COVID-19 test not required for:<br>o Weddings with fewer than 30 guests<br>o Meetings and conferences not exceeding 30% of capacity<br>· Movements are prohibited from 10pm to 5am	6
2020-10-13	· Offices of public and private institutions will operate at 50% capacity<br>· Places of worship will increase to 50% of venue capacity<br>· Church wedding ceremonies and funeral gatherings to not exceed 75 persons	7
2020-11-12	· Gyms and swimming pools to resume activities<br>· Live performances and cultural shows will resume activities	8
2020-12-15	· From 15-21/12/2020, movements are prohibited from 9pm to 4am<br>· From 22-12/2020 – 4/01/2021, movements are prohibited from 8pm to 4am<br>· All social gatherings including wedding ceremonies and celebrations of all kinds are prohibited both in public and private settings<br>· Offices of public and private institutions will operate at 30% capacity<br>· All gyms and swimming pools are closed	9

2021-01-05	· All business establishments, including restaurants, shops, markets, and malls will close operations daily by 6pm<br>· Movements are prohibited from 8pm to 4am<br>· Public and private transports are prohibited to and from Kigali<br>· Domestic and international tourists may travel across districts but must possess a negative COVID-19 test	10
2021-01-18	In Kigali:<br>· Unnecessary movements outside the home are prohibited<br>· Travel between Kigali and other provinces is not permitted<br>· Businesses are closed<br>· Schools, universities and places of worship closed<br><br>Elsewhere:<br>· Movements are prohibited from 6pm to 4am<br>· Schools are open	11
2021-02-02	In Kigali:<br>· Movements are prohibited from 7pm to 4am<br>· Markets and malls are open for essential traders, mayt not exceed 50% capacity, and must close by 5pm<br>· Transport within Kigali permitted, but not between Kigali and other regions<br>Elsewhere:<br>· Movements are prohibited between 7pm and 4am	12

Sources:

1. https://www.primature.gov.rw/index.php?id=43&no_cache=1&L=..%2F&tx_drblob_pi1%5BdownloadUid%5D=784
2. https://www.primature.gov.rw/index.php?id=43&no_cache=1&L=..%2F&tx_drblob_pi1%5BdownloadUid%5D=786

3. https://www.primature.gov.rw/index.php?id=43&no_cache=1&L=..%2F&tx_drblob_pi1%5BdownloadUid%5D=801
4. https://www.primature.gov.rw/index.php?id=43&no_cache=1&L=..%2F&tx_drblob_pi1%5BdownloadUid%5D=803
5. https://www.primature.gov.rw/index.php?id=43&no_cache=1&L=..%2F&tx_drblob_pi1%5BdownloadUid%5D=806
6. https://www.primature.gov.rw/index.php?id=43&no_cache=1&L=..%2F&tx_drblob_pi1%5BdownloadUid%5D=810

7. https://www.primature.gov.rw/index.php?id=43&no_cache=1&L=..%2F&tx_drblob_pi1%5BdownloadUid%5D=820
8. https://www.primature.gov.rw/index.php?id=43&no_cache=1&L=..%2F&tx_drblob_pi1%5BdownloadUid%5D=829
9. https://www.primature.gov.rw/index.php?id=43&no_cache=1&L=..%2F&tx_drblob_pi1%5BdownloadUid%5D=843
10. https://www.primature.gov.rw/index.php?id=43&no_cache=1&L=540&tx_drblob_pi1%5BdownloadUid%5D=846
11. https://www.primature.gov.rw/index.php?id=43&no_cache=1&tx_drblob_pi1%5BdownloadUid%5D=849
12. https://www.primature.gov.rw/index.php?id=43&no_cache=1&tx_drblob_pi1%5BdownloadUid%5D=852

Supplementary Table S2. GISAID accession identifiers for the Rwandan genomes in this study for which individual travel history metadata are available. We list the collection date for each GISAID entry, along with the country from which the infected traveller returned to Rwanda, and the subtree assignment based on Supplementary Figure S3.

Accession ID	Collection date	Travel history	Subtree
EPI_ISL_707771	2020-06-16	Tanzania	A
EPI_ISL_707772	2020-06-16	Tanzania	A
EPI_ISL_925865	2020-12-17	China	A
EPI_ISL_1063905	2020-12-14	Uganda	A
EPI_ISL_925848	2020-12-14	Kenya	A
EPI_ISL_925851	2020-12-15	Kenya	A

EPI_ISL_1064164	2021-01-4	Tanzania	A
EPI_ISL_1064163	2021-01-4	Tanzania	A
EPI_ISL_925850	2020-12-16	Uganda	A
EPI_ISL_1064154	2021-01-5	Kenya	A
EPI_ISL_707712	2020-06-8	South Sudan	A
EPI_ISL_735448	2020-10-19	Morocco	B.1
EPI_ISL_960250	2020-08-28	Tanzania	B.1
EPI_ISL_735445	2020-10-22	Italy	B.1
EPI_ISL_925896	2020-12-15	Democratic Republic of the Congo	B.1
EPI_ISL_1063900	2020-10-18	Kenya	B.1
EPI_ISL_707789	2020-10-19	Uganda	B.1

Supplementary Table S3. Posterior support for transitions into Rwanda and corresponding mean number of introductions, as estimated using Markov jumps. For each subtree analysis, only transition rates with a Bayes factor above 3 are presented.

From	Subtree	Bayes factor	Posterior probability	Mean Markov jumps
Uganda	A	63104	1	13.1
Tanzania	A	63104	>0.99	2.6
Kenya	A	2396.3	0.99	4.4
South Sudan	A	242.5	0.88	1.1
China	A	234.4	0.88	1.0
Nigeria	A	14.5	0.31	0.4
Denmark	A	7.6	0.19	0.2
Italy	B.1	657.2	0.95	1.6
Uganda	B.1	469.0	0.93	1.5
Kenya	B.1	208.4	0.85	3.2
Tanzania	B.1	195.1	0.84	1.2
Morocco	B.1	156.5	0.81	1.1
Democratic Republic of the Congo	B.1	49.7	0.58	1.1

Supplementary Table S4. List of sequencing primers and oligonucleotides list.

name	pool	seq
SARSCoV2120_1_LEFT	nCov-2019_1	ACCAACCAACTTTCGATCTCTTGT
SARSCoV2120_1_RIGHT	nCov-2019_1	GGTTGCATTCATTTGGTGACGC
SARSCoV2120_3_LEFT	nCov-2019_1	GGCTTGAAGAGAAGTTTAAGGAAGGT
SARSCoV2120_3_RIGHT	nCov-2019_1	GATTGTCCTCACTGCCGTCTTG
SARSCoV2120_5_LEFT	nCov-2019_1	ACCTACTAAAAAGGCTGGTGGC
SARSCoV2120_5_RIGHT	nCov-2019_1	AGCATCTTGTAGAGCAGGTGGA
SARSCoV2120_7_LEFT	nCov-2019_1	ACCTGGTGTATACGTTGTCTTTGG
SARSCoV2120_7_RIGHT	nCov-2019_1	GCTGAAATCGGGGCCATTTGTA
SARSCoV2120_9_LEFT	nCov-2019_1	AGAAGTTACTGGCGATAGTTGTAATAACT
SARSCoV2120_9_RIGHT	nCov-2019_1	TGCTGATATGTCCAAAGCACCA
SARSCoV2120_11_LEFT	nCov-2019_1	AGACACCTAAGTATAAGTTTGTTCGCA

SARSCoV2120_11_RIGHT	nCov-2019_1	GCCCACATGGAAATGGCTTGAT
SARSCoV2120_13_LEFT	nCov-2019_1	ACCTCTTACAACAGCAGCCAAAC
SARSCoV2120_13_RIGHT	nCov-2019_1	CGTCCTTTTCTTGGAAGCGACA
SARSCoV2120_15_LEFT	nCov-2019_1	TTTTAAGGAATTACTTGTGTATGCTGCT
SARSCoV2120_15_RIGHT	nCov-2019_1	ACACACAACAGCATCGTCAGAG
SARSCoV2120_17_LEFT	nCov-2019_1	TCAAGCTTTTTGCAGCAGAAACG
SARSCoV2120_17_RIGHT	nCov-2019_1	CCAAGCAGGGTTACGTGTAAGG
SARSCoV2120_19_LEFT	nCov-2019_1	GGCACATGGCTTTGAGTTGACA
SARSCoV2120_19_RIGHT	nCov-2019_1	CCTGTTGTCCATCAAAGTGTCCC
SARSCoV2120_21_LEFT	nCov-2019_1	TCTGTAGTTTCTAAGGTTGTCAAAGTGA
SARSCoV2120_21_RIGHT	nCov-2019_1	GCAGGGGGTAATTGAGTTCTGG
SARSCoV2120_23_LEFT	nCov-2019_1	ACTTTAGAGTCCAACCAACAGAATCT
SARSCoV2120_23_RIGHT	nCov-2019_1	TGACTAGCTACACTACGTGCCC

SARSCoV2120_25_LEFT	nCov-2019_1	TGCTGCTACTAAAATGTCAGAGTGT
SARSCoV2120_25_RIGHT	nCov-2019_1	CATTTCCAGCAAAGCCAAAGCC
SARSCoV2120_27_LEFT	nCov-2019_1	TGGATCACCGGTGGAATTGCTA
SARSCoV2120_27_RIGHT	nCov-2019_1	TGTTCGTTTAGGCGTGACAAGT
SARSCoV2120_29_LEFT	nCov-2019_1	TGAGGGAGCCTTGAATACACCA
SARSCoV2120_29_RIGHT	nCov-2019_1	TAGGCAGCTCTCCCTAGCATTG
SARSCoV2120_2_LEFT	nCov-2019_2	CCATAATCAAGACTATTCAACCAAGGGT
SARSCoV2120_2_RIGHT	nCov-2019_2	ACAGGTGACAATTTGTCCACCG
SARSCoV2120_4_LEFT	nCov-2019_2	GGAATTTGGTGCCACTTCTGCT
SARSCoV2120_4_RIGHT	nCov-2019_2	CCTGACCCGGGTAAGTGGTTAT
SARSCoV2120_6_LEFT	nCov-2019_2	ACTTCTATTAAATGGGCAGATAACAACTG
SARSCoV2120_6_RIGHT	nCov-2019_2	GATTATCCATTCCCTGCGCGTC
SARSCoV2120_8_LEFT	nCov-2019_2	CAATCATGCAATTGTTTTTCAGCTATTTTG

SARSCoV2120_8_RIGHT	nCov-2019_2	TGACTTTTTGCTACCTGCGCAT
SARSCoV2120_10_LEFT	nCov-2019_2	TTTACCAGGAGTTTTCTGTGGTGT
SARSCoV2120_10_RIGHT	nCov-2019_2	TGGGCCTCATAGCACATTGGTA
SARSCoV2120_12_LEFT	nCov-2019_2	ATGGTGCTAGGAGAGTGTGGAC
SARSCoV2120_12_RIGHT	nCov-2019_2	GGATTTCCCACAATGCTGATGC
SARSCoV2120_14_LEFT	nCov-2019_2	ACAGGCACTAGTACTGATGTCGT
SARSCoV2120_14_RIGHT	nCov-2019_2	GTGCAGCTACTGAAAAGCACGT
SARSCoV2120_16_LEFT	nCov-2019_2	ACAACACAGACTTTATGAGTGTCTCT
SARSCoV2120_16_RIGHT	nCov-2019_2	CTCTGTCAGACAGCACTTCACG
SARSCoV2120_18_LEFT	nCov-2019_2	GCACATAAAGACAAATCAGCTCAATGC
SARSCoV2120_18_RIGHT	nCov-2019_2	TGTCTGAAGCAGTGGAAAAGCA
SARSCoV2120_20_LEFT	nCov-2019_2	ACAATTTGATACTTATAACCTCTGGAACAC
SARSCoV2120_20_RIGHT	nCov-2019_2	GATTAGGCATAGCAACACCCGG

SARSCoV2120_22_LEFT	nCov-2019_2	GTGATGTTCTTGTTAACAACTAAACGAACA
SARSCoV2120_22_RIGHT	nCov-2019_2	AACAGATGCAAATCTGGTGGCG
SARSCoV2120_24_LEFT	nCov-2019_2	GCTGAACATGTCAACAACTCATATGA
SARSCoV2120_24_RIGHT	nCov-2019_2	ATGAGGTGCTGACTGAGGGAAG
SARSCoV2120_26_LEFT	nCov-2019_2	GCCTTGAAGCCCCTTTTCTCTA
SARSCoV2120_26_RIGHT	nCov-2019_2	AATGACCACATGGAACGCGTAC
SARSCoV2120_28_LEFT	nCov-2019_2	TTTGTGCTTTTTAGCCTTTCTGCT
SARSCoV2120_28_RIGHT	nCov-2019_2	GTTTGGCCTTGTTGTTGTTGGC

Supplementary Figures

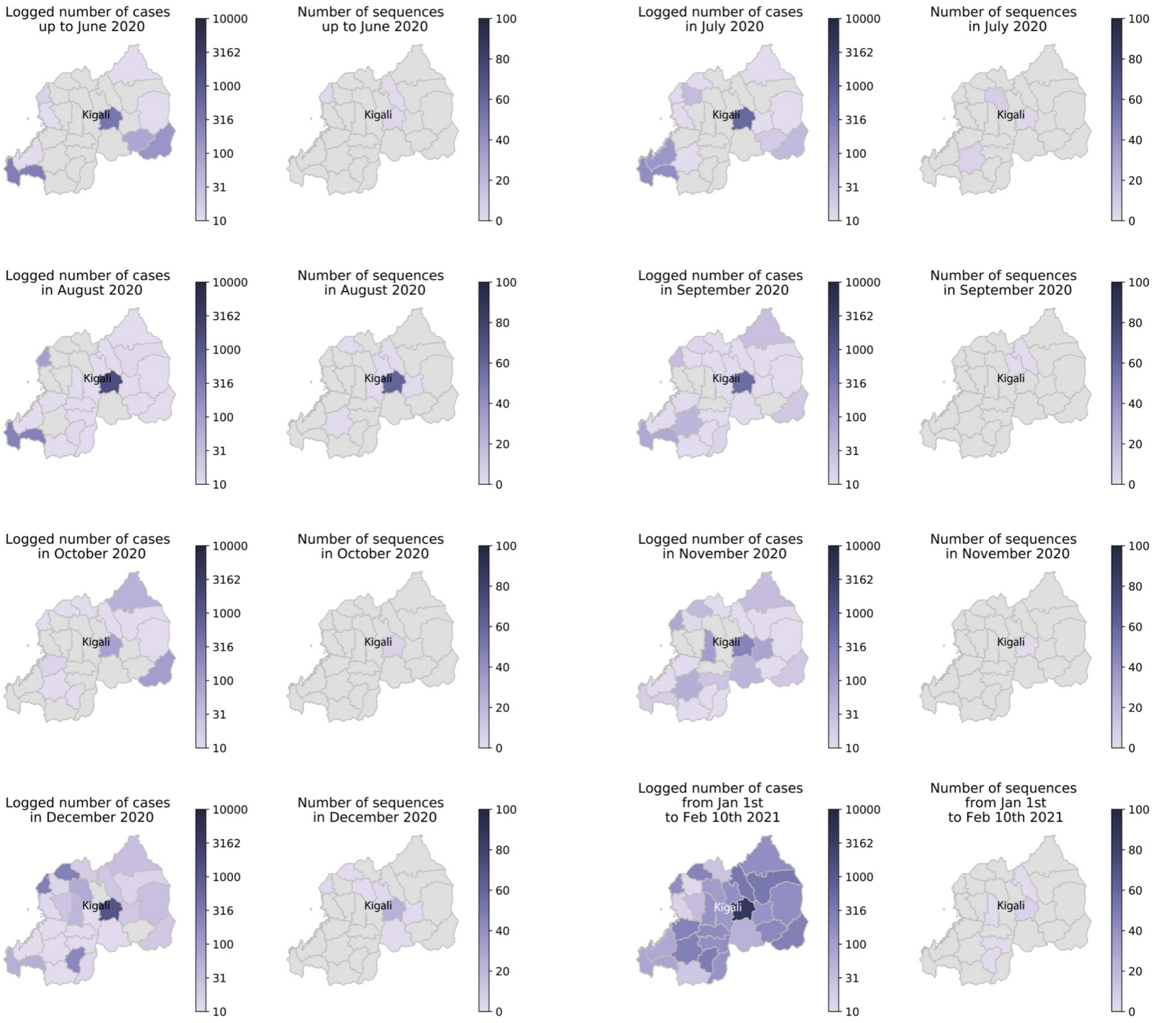

Supplementary Figure S1. Maps showing case and sequence counts for each region and for each month in the data set. Note that the first set of maps show all data until the end of June 2020, and the last map has data from the 1st of January to the 10th of February. Kigali, the capital city of Rwanda, and where most cases and sequences are from, is indicated on each map.

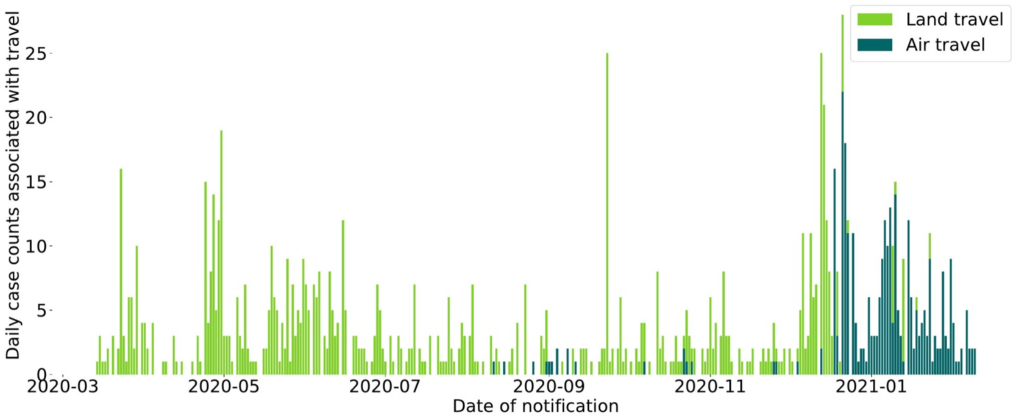

Supplementary Figure S2. Timeline showing the number of cases with direct travel history. Light green are those cases who entered Rwanda by land, and dark green are those who entered by air. Note that the air borders were closed from 2020-03-22 to 2020-07-30 (see Supplementary Table S2). Of the 54 countries that 424 air passengers originated in, 60 were from Tanzania (14%), 54 from Kenya (13%), 38 from Uganda (9%), 30 from Burundi (7%), 27 from Nigeria (6%), 23 from India (5%), 19 from Dubai (4%), 14 from Cameroon (3%), 13 from the USA (3%) and 11 from the DRC (3%). The remaining countries had 10 passengers or fewer, and we provide a complete overview of the incoming travellers who entered Rwanda by air in a Supplementary File.

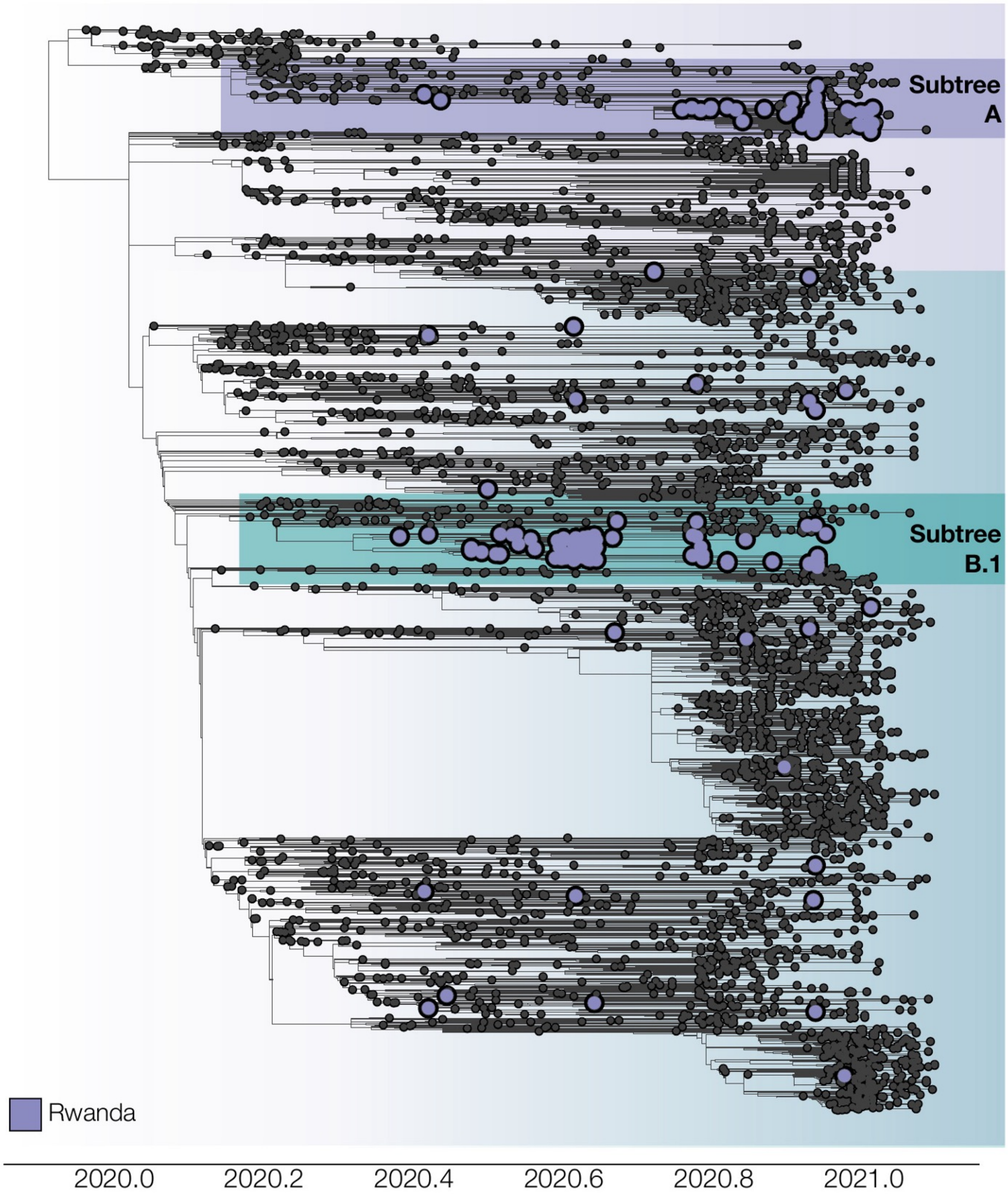

Supplementary Figure S3. Time-calibrated phylogenetic tree of Rwandan SARS-CoV-2 genome sequences in the global context of the current SARS-CoV-2 pandemic. Two large clusters of Rwandan sequences can be identified, representing lineages A.23.1 and B.1.380, with other individual Rwandan sequences scattered throughout the phylogeny. Subtrees encompassing

lineages A.23.1 and B.1.380 (referred to as subtree A and subtree B.1) were selected to perform Bayesian phylogeographic reconstruction that accommodates individual travel histories. Two variants of concern (VOC) sequences were detected during sequencing: one from lineage B.1.1.7, a returning traveller from Burundi, and one from lineage B.1.351, a returning traveller from the Democratic Republic of the Congo.

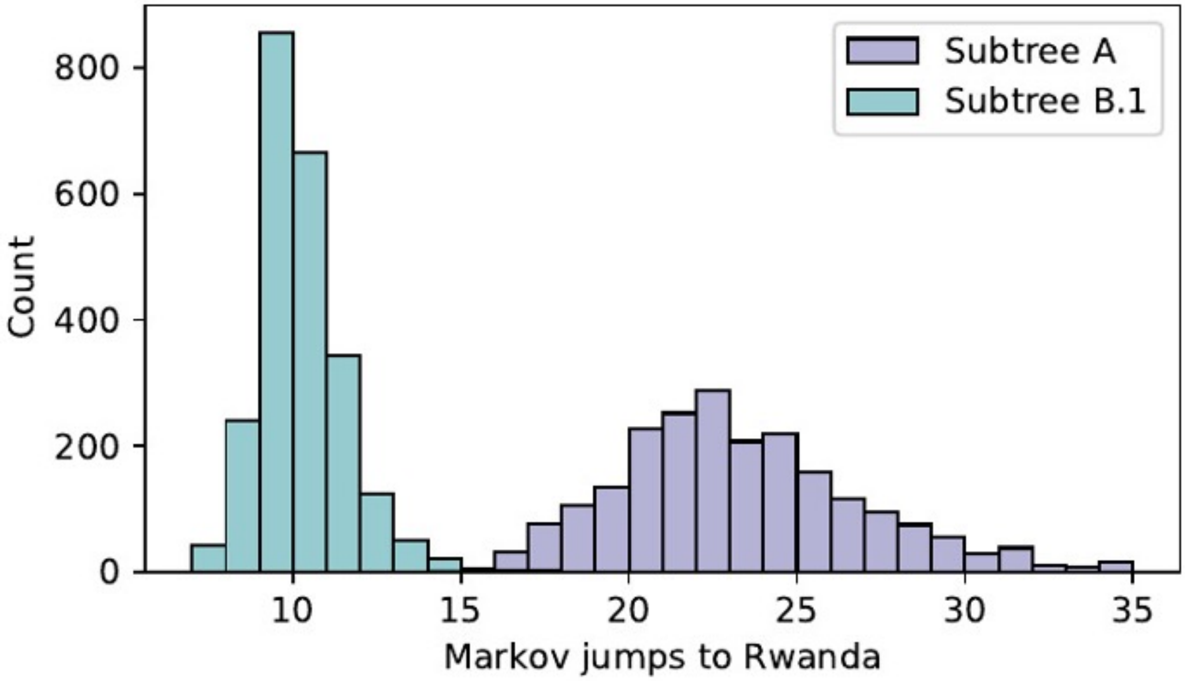

Supplementary Figure S4. Posterior number of introductions into Rwanda for subtrees A and B.1. The total number of Markov jumps into Rwanda for each subtree was estimated via stochastic mapping on an asymmetric discrete state phylogeographic model. Despite the lower number of Rwandan sequences (subtree A: 49; subtree B: 134), subtree A reveals a higher number of introduction events (mean=22.8; 95%HPD=[16-29]) compared to subtree B.1 (mean=9.8; 95%HPD=[8-12]).

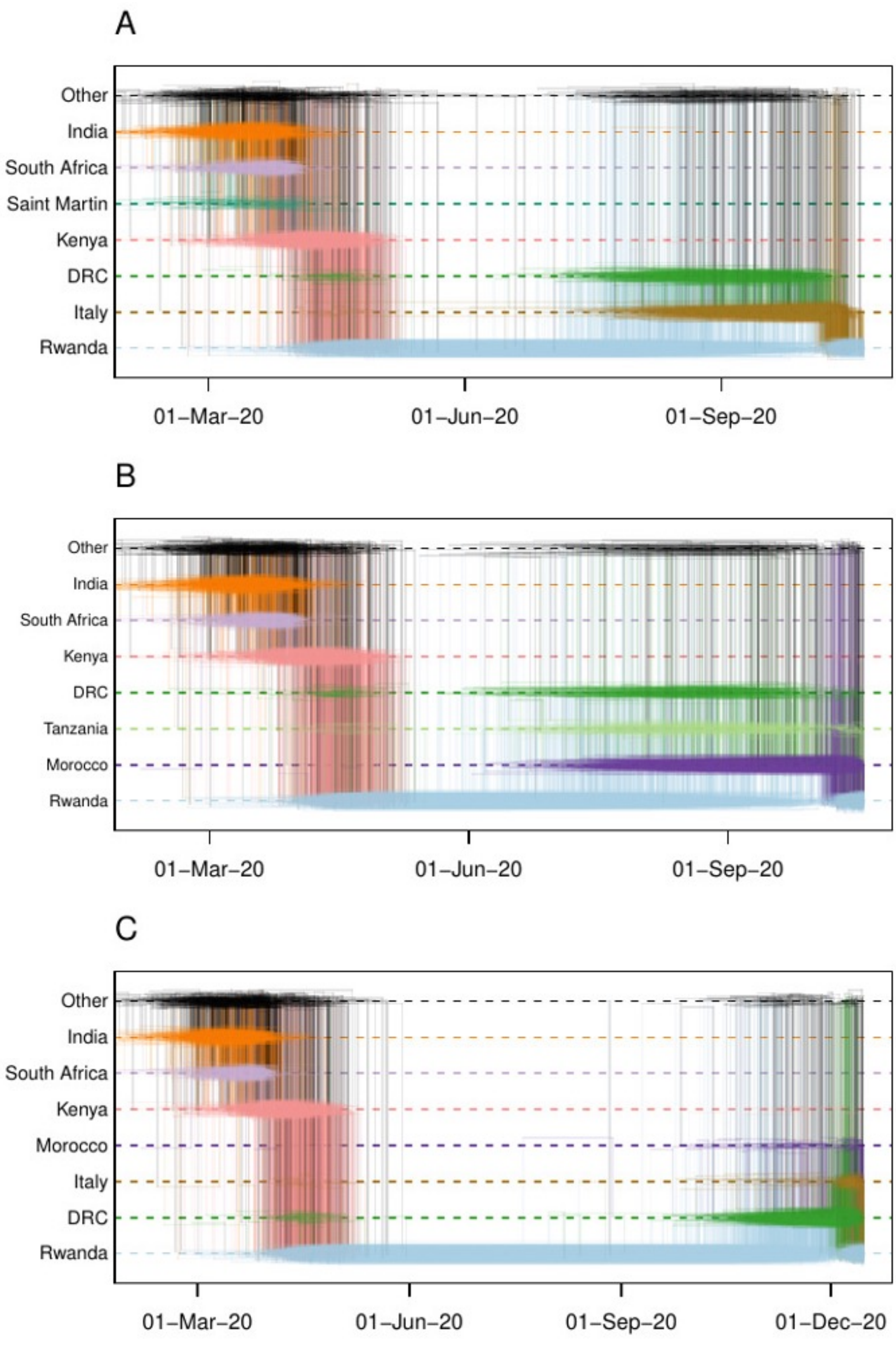
A
Other
India
South Africa
Saint Martin
Kenya
DRC
Italy
Rwanda
01−Mar−20
01−Jun−20
01−Sep−20
B
Other
India
South Africa
Kenya
DRC
Tanzania
Morocco
Rwanda
01−Mar−20
01−Jun−20
01−Sep−20
C
Other
India
South Africa
Kenya
Morocco
Italy
DRC
Rwanda
01−Mar−20
01−Jun−20
01−Sep−20
01−Dec−20

Supplementary Figure S5. Markov jump trajectory plots for three selected Rwandan infected individuals with travel history (returning) from Italy (A), Morocco (B) and the Democratic Republic of the Congo (C). Similar to Figure 8 (panel C) in the main text, the ancestral histories inferred for these three isolates show support for a bidirectional flow of viral lineages between each corresponding travel location and Rwanda.

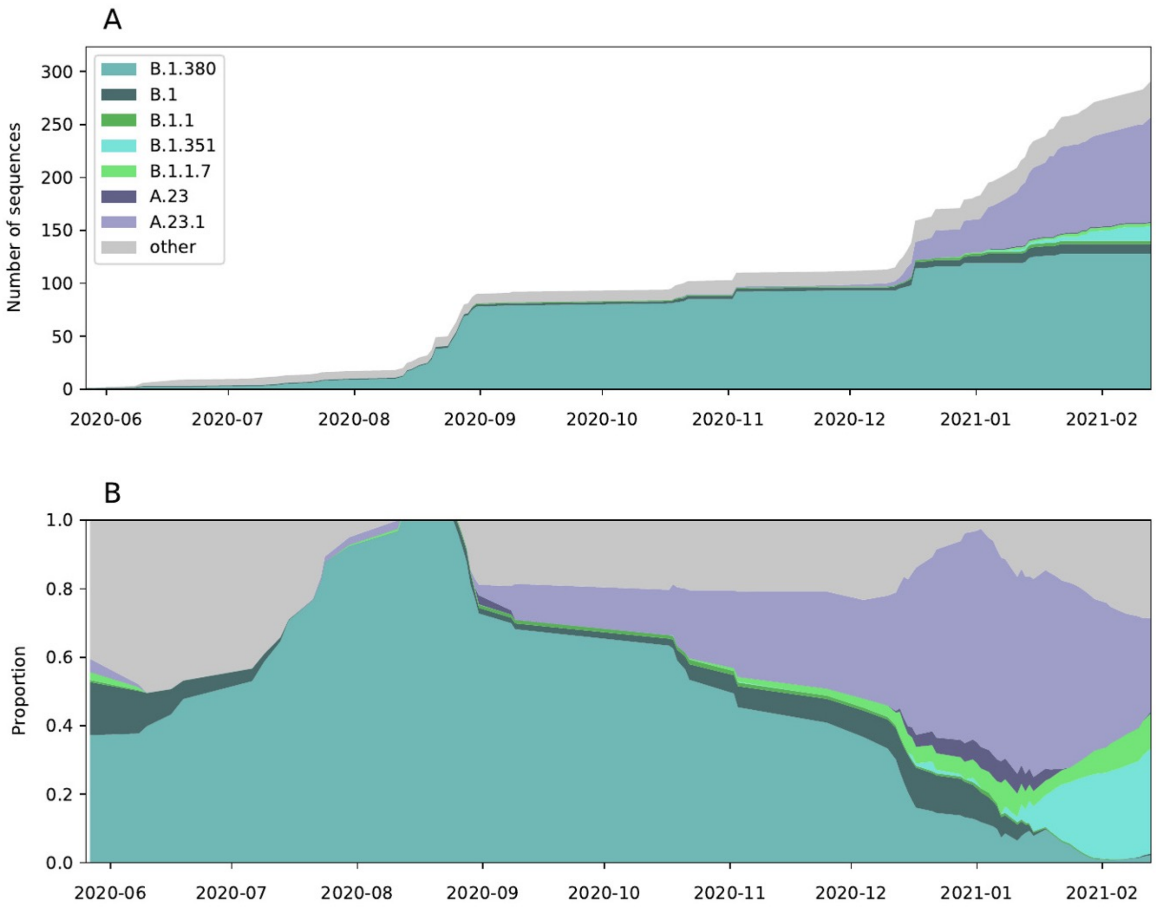

Supplementary Figure S6. Lineage frequency plots for Rwanda. A) Cumulative number over time of Rwandan SARS-CoV-2 sequences by lineage. B) Frequencies over time of the key lineages identified in this study, along with the B.1.1.7 and B.1.351 variants of concern. Regarding the A.23.1 and B.1.380 lineages in this study, we note that the introduction of B.1.380 in Rwanda was followed by its rise to dominance by the start of July, 2020. By the start of September, 2020, B.1.380 incidence started to decline at the same time as the increase of several lineages including that of A.23.1, which rose to dominance by the end of 2020. Lineage B.1.380 had almost entirely disappeared by the end of our study, with A.23.1, B.1.351 and B.1.1.7

causing the majority of infections. Note that because of the limited number of genomes available, the estimated frequencies over time are sensitive to low lineage counts.

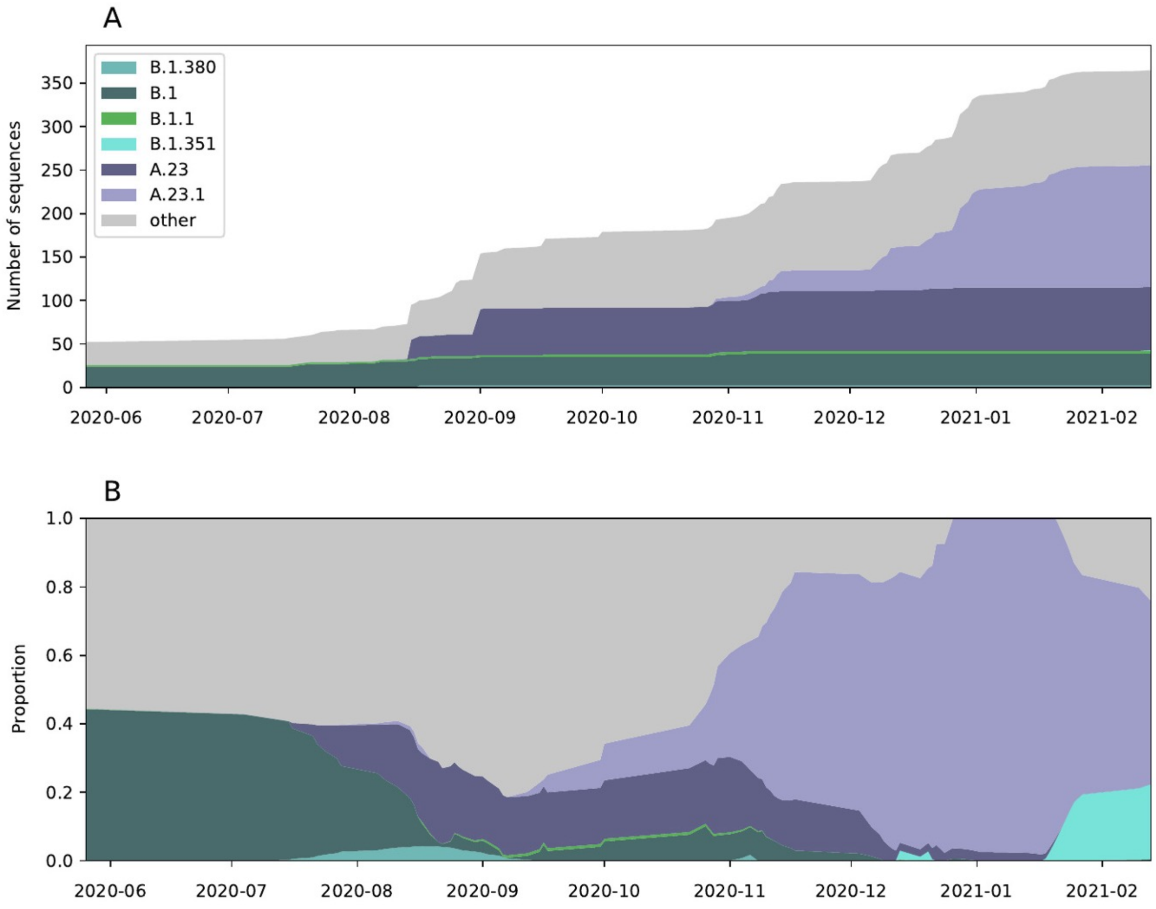

Supplementary Figure S7. Lineage frequency plots for Uganda. A) Cumulative number over time of Ugandan SARS-CoV-2 sequences by lineage. B) Frequencies over time of the key lineages identified in this study, along with the B.1.351 variant of concern. B.1.380 is barely found in Uganda, with only a few genomes being available from the summer of 2020. At the start of September, 2020, A.23.1 started its rapid increase toward being the dominant lineage in Uganda, having evolved from the A.23 lineage that remained responsible for a sizable number of infections after the emergence of A.23.1. By the end of our study period, A.23.1 was still the dominant lineage, although the B.1.351 variant of concern had been increasing in frequency for the past month. Note that because of the limited number of genomes available, the estimated frequencies over time are sensitive to low lineage counts.

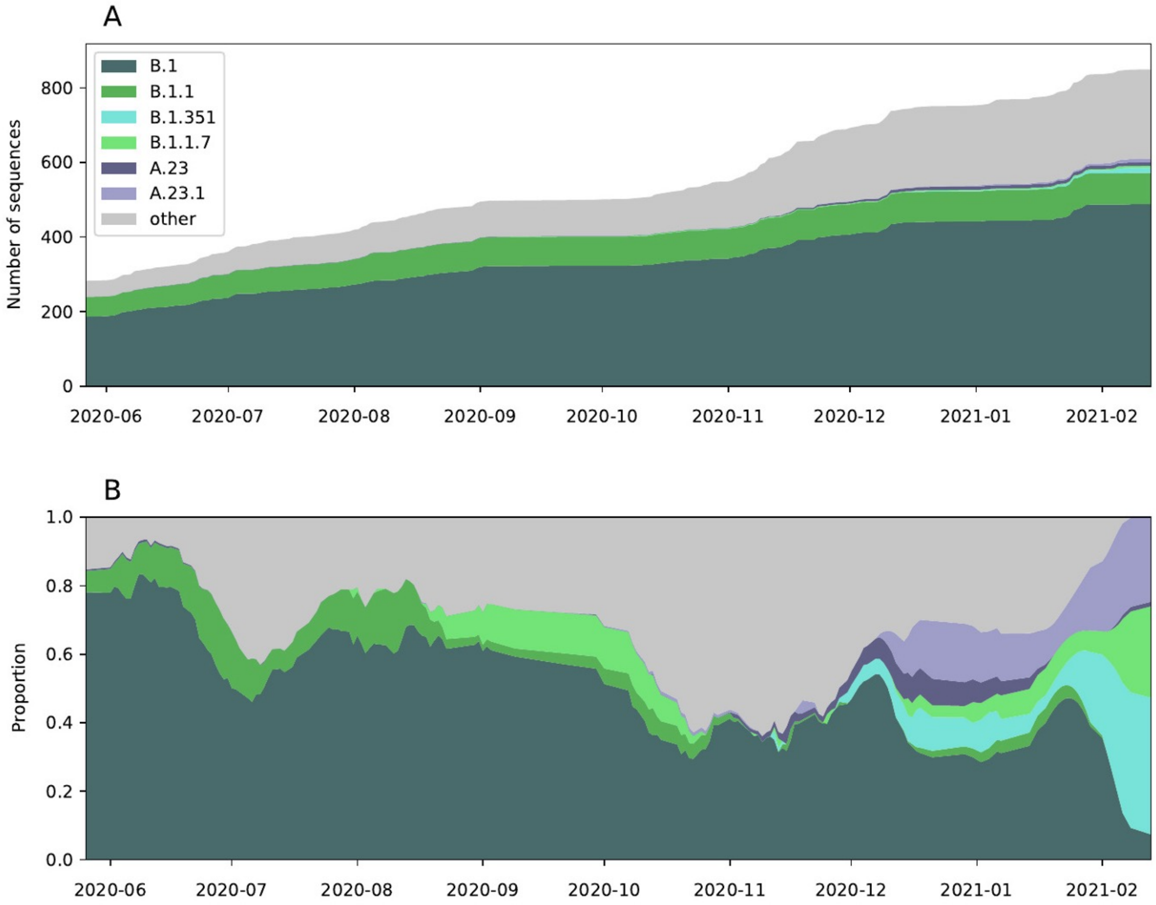

Supplementary Figure S8. Lineage frequency plots for Kenya. A) Cumulative number over time of Kenyan SARS-CoV-2 sequences by lineage. B) Frequencies over time of the key lineages identified in this study, along with the B.1.1.7 and B.1.351 variants of concern. Since the start of the pandemic, lineage B.1 was responsible for most of the infections, with an increase of lineage B.1.351 (and to a lesser extent B.1.1.7) towards the end of our study period. Lineage B.1.380 is not found in Kenya, whereas lineage A.23.1 started to increase in frequency since the start of December, 2020. Note that because of the limited number of genomes available, the estimated frequencies over time are sensitive to low lineage counts. This is best seen during September and October of 2020, with one single case of B.1.1.7 in each of those months making it seem as if this lineage represented a sizable proportion of the epidemic in Kenya.

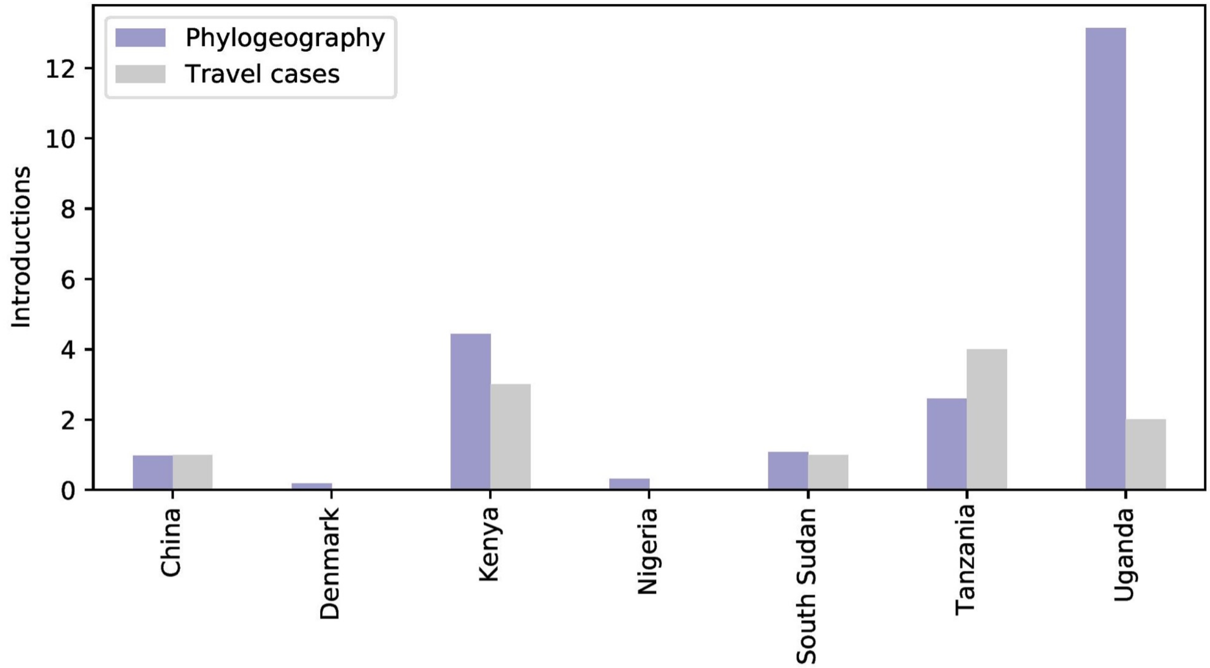

Supplementary Figure S9. Subtree A introductions into Rwanda by country of origin and travel cases. The posterior mean number of Markov jumps obtained from the phylogeographic analysis for lineage A.23.1 is plotted against the number of travel-associated cases from each location. Only countries of origin with supported rates (Bayes factor >3) are shown. The phylogeographic analysis is able to identify multiple introductions from Uganda and Kenya not accounted for in the available travel records. However, in the case of Tanzania, the phylogeographic reconstruction shows a lower inferred number of introduction events (2.6) than the number of recorded travel cases (4). This can be explained by the fact that the Tanzanian sequences with associated travel dates are clustered in two pairs with identical sequences and sampling dates.

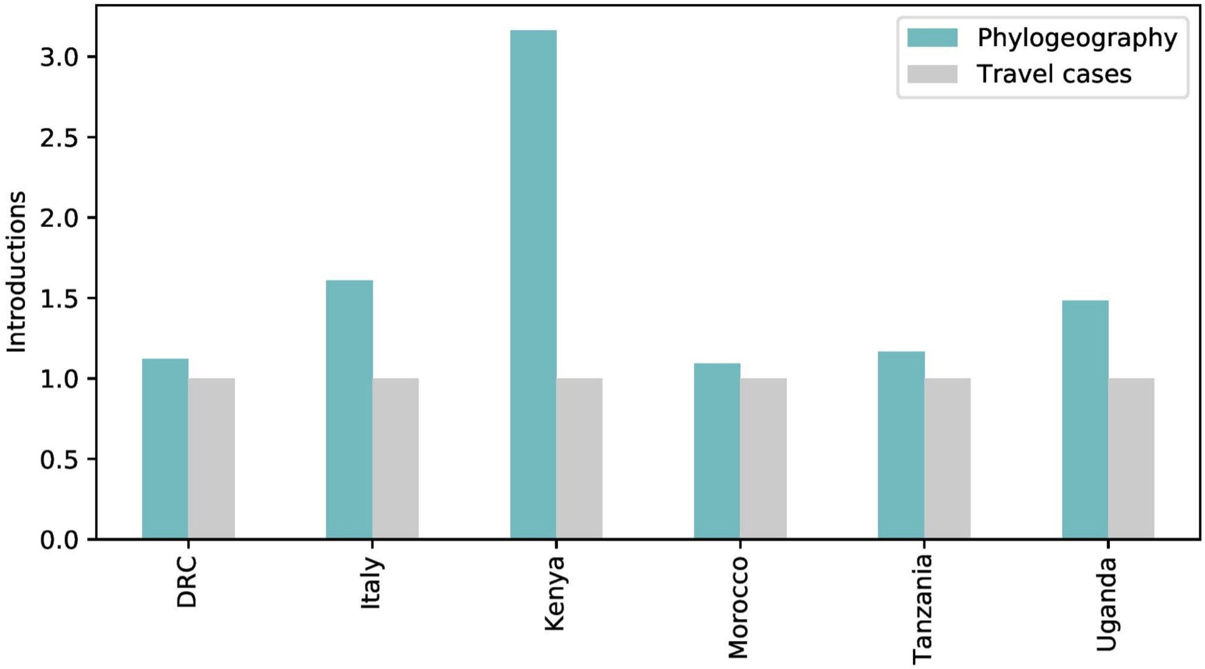

Supplementary Figure S10. Subtree B.1 introductions into Rwanda by country of origin and travel cases. The posterior mean number of Markov jumps obtained from the phylogeographic analysis for lineage B.1.380 is plotted against the number of travel associated cases from each location. Only countries of origin with supported rates (Bayes factor >3) are shown. The phylogeographic analysis is able to identify multiple introductions from Kenya not accounted for in the travel cases available.

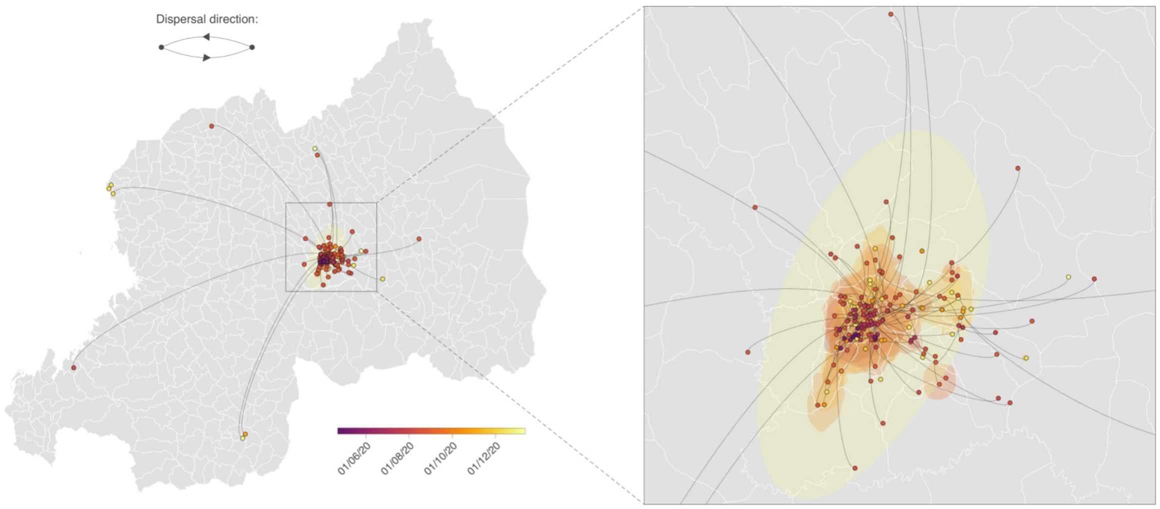

Supplementary Figure S11. Spatially-explicit phylogeographic reconstruction of the dispersal history of SARS-CoV-2 lineages sampled in Rwanda. Spatially-explicit phylogeographic reconstruction was performed along the Rwandan clades identified within the two subtrees A and B.1. For each clade we mapped the maximum clade credibility (MCC) tree and overall 80% highest posterior density (HPD) regions reflecting the uncertainty related to the phylogeographic inference. MCC trees and 80% HPD regions are based on 1,000 trees subsampled from each post burn-in posterior distribution. MCC tree nodes were coloured according to their time of occurrence, and 80% HPD regions were computed for successive time layers and then superimposed using the same colour scale reflecting time. Continuous phylogeographic reconstructions were only performed along clades linking at least three sequences sampled in Rwanda and for which the sector of origin was known. Besides the phylogenetic branches of MCC trees obtained by continuous phylogeographic inference, we also mapped sampled sequences belonging to clades linking less than three geo-referenced sequences. The most precise geographic locations associated with the Rwandan genomes were sectors (see e.g. https://en.wikipedia.org/wiki/Sectors_of_Rwanda), which are relatively large administrative polygons, from which a point was randomly sampled for this visualisation. Sector borders are represented by white lines.

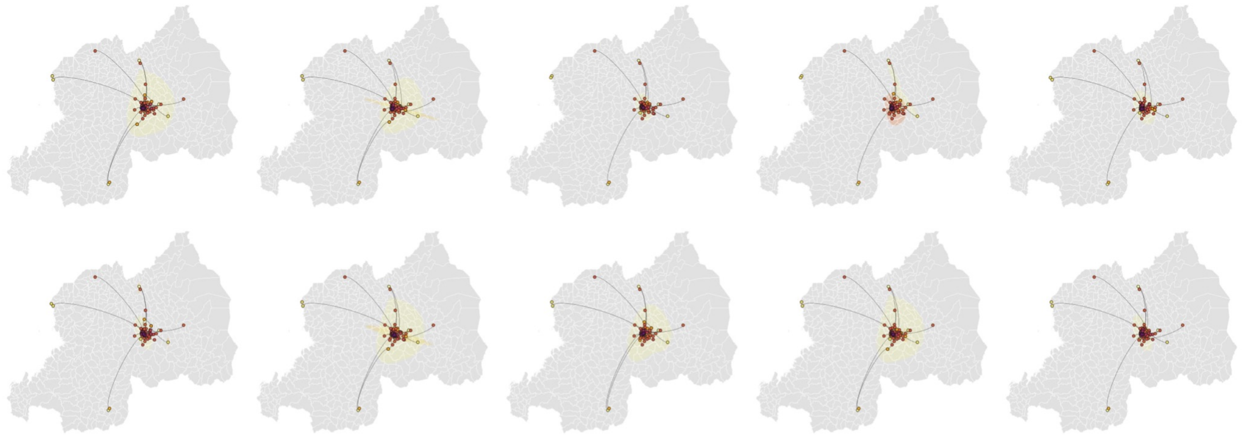

Supplementary Figure S12. Subsampled continuous phylogeographic reconstructions. Ten replicates of our continuous phylogeographic reconstructions based on subsets of sequences

obtained by randomly selecting a maximum of two sequences per administrative “sector” area. We refer to the legend of Supplementary Figure S11 for additional details on the mapping of this continuous phylogeographic inference.

Supplementary References

Daniel Lule Bugembe, My V. T. Phan, Isaac Ssewanyana, Patrick Semanda, Hellen Nansumba, Beatrice Dhaala, Susan Nabadda, Áine Niamh O'Toole, Andrew Rambaut, Pontiano Kaleebu, Matthew Cotten. 2021. Emergence and spread of a SARS-CoV-2 lineage A variant (A.23.1) with altered spike protein in Uganda. Nature Microbiology 6: 1094-1101.

Simon Dellicour, Cécile Troupin, Fatemeh Jahanbakhsh, Akram Salama, Siamak Massoudi, Madjid K. Moghaddam, Guy Baele, Philippe Lemey, Alireza Gholami, Hervé Bourhy. 2019. Using phylogeographic approaches to analyse the dispersal history, velocity and direction of viral lineages - Application to rabies virus spread in Iran. Molecular Ecology 28(18): 4335-4350.

Simon Dellicour, Keith Durkin, Samuel L. Hong, Bert Vanmechelen, Joan Martí-Carreras, Mandev S. Gill, Cécile Meex, Sébastien Bontems, Emmanuel André, Marius Gilbert, Conor Walker, Nicola De Maio, Nuno R. Faria, James Hadfield, Marie-Pierre Hayette, Vincent Bours, Tony Wawina-Bokalanga, Maria Artesi, Guy Baele, Piet Maes. 2021. A Phylodynamic Workflow to Rapidly Gain Insights into the Dispersal History and Dynamics of SARS-CoV-2 Lineages. Molecular Biology and Evolution 38(4): 1608-1613.

Louis du Plessis, John T. McCrone, Alexander E. Zarebski, Verity Hill, Christopher Ruis, Bernardo Gutierrez, Jayna Raghwani, Jordan Ashworth, Rachel Colquhoun, Thomas R. Connor, Nuno R. Faria, Ben Jackson, Nicholas J. Loman, Áine O'Toole, Samuel M. Nicholls, Kris V. Parag, Emily Scher, Tetyana I. Vasylyeva, Erik M. Volz, Alexander Watts, Isaac I. Bogoch, Kamran Khan, COVID-19 Genomics UK (COG-UK) Consortium, David M. Aanensen, Moritz U. G. Kraemer, Andrew Rambaut, Oliver G. Pybus. 2021. Establishment and lineage dynamics of the SARS-CoV-2 epidemic in the UK. Science 12, 371(6530):708-712.

Nikki E Freed, Markéta Vlková, Muhammad B Faisal, Olin K Silander. Rapid and inexpensive whole-genome sequencing of SARS-CoV-2 using 1200 bp tiled amplicons and Oxford Nanopore Rapid Barcoding. 2020. Biology Methods and Protocols 5(1): bpaa014.

George Githinji, Zaydah R. de Laurent, Khadija Said Mohammed, Donwilliams O. Omuoyo, Peter M. Macharia, John M. Morobe, Edward Otieno, Samson M. Kinyanjui, Ambrose Agweyu, Eric Maitha, Ben Kitole, Thani Suleiman, Mohamed Mwakinangu, John Nyambu, John Otieno, Barke Salim, Kadondi Kasera, John Kiiru, Rashid Aman, Edwine Barasa, George Warimwe, Philip Bejon, Benjamin Tsofa, Lynette Isabella Ochola-Oyier, D. James Nokes, Charles N. Agoti. 2020. Tracking the introduction and spread of SARS-CoV-2 in coastal Kenya. medRxiv 2020.10.05.20206730.

Antanas Kalkauskas, Umberto Perron, Yuxuan Sun, Nick Goldman, Guy Baele, Stephane Guindon, Nicola De Maio. 2021. Sampling bias and model choice in continuous phylogeography: Getting lost on a random walk. PLoS Computational Biology 17(1): e1008561.

Philippe Lemey, Samuel L. Hong, Verity Hill, Guy Baele, Chiara Poletto, Vittoria Colizza, Áine O'Toole, John T. McCrone, Kristian G. Andersen, Michael Worobey, Martha I. Nelson, Andrew Rambaut & Marc A. Suchard. 2020. Accommodating individual travel history and unsampled diversity in Bayesian phylogeographic inference of SARS-CoV-2. Nature Communications 11: 5110.

Abraham Savitzky, Marcel J. E. Golay. 1964. Smoothing and Differentiation of Data by Simplified Least Squares Procedures. Analytical Chemistry 36: 1627-1639.

Pauli Virtanen, Ralf Gommers, Travis E. Oliphant, Matt Haberland, Tyler Reddy, David Cournapeau, Evgeni Burovski, Pearu Peterson, Warren Weckesser, Jonathan Bright, Stéfan J. van der Walt, Matthew Brett, Joshua Wilson, K. Jarrod Millman, Nikolay Mayorov, Andrew R. J. Nelson, Eric Jones, Robert Kern, Eric Larson, C J Carey, İlhan Polat, Yu Feng, Eric W. Moore, Jake VanderPlas, Denis Laxalde, Josef Perktold, Robert Cimrman, Ian Henriksen, E. A. Quintero, Charles R. Harris, Anne M. Archibald, Antônio H. Ribeiro, Fabian Pedregosa, Paul van Mulbregt & SciPy 1.0 Contributors. 2020. SciPy 1.0: fundamental algorithms for scientific computing in Python. Nature Methods 17: 261-272.
