# Supplementary material for: Genomic sequencing of SARS-CoV-2 in Rwanda reveals the importance of incoming travelers on lineage diversity
Source: Nat Commun. 2021 Sep 29;12:5705. doi: 10.1038/s41467-021-25985-7 (PMC8481346; doi:10.1038/s41467-021-25985-7)
Supplement: Supplementary file 4 — Description of Additional Supplementary Files [file 41467_2021_25985_MOESM4_ESM.pdf]

Description of Additional Supplementary Files

File Name: Supplementary Data 1
Description: de-identified master metadata of the 203 SARS-CoV-2 genomes analyzed in this study.

File Name: Supplementary Data 2
Description: gisaid_hcov-19_acknowledgement_table_2021_05_26_07.pdf: GISAID acknowledgement table, as generated through the GISAID website, illustrating the contributions of both the submitting and the originating laboratories in generating the SARS-CoV-2 genome sequences used in this study.

File Name: Supplementary Data 3
Description: subtreeA_travelHist_noAln.xml: BEAST 1.10.5 XML file to perform travel history-aware discrete Bayesian phylogeographic analysis, on subtree A of the full data set (see main text for the description of subtree A).

File Name: Supplementary Data 4
Description: subtreeB_travelHist_noAln.xml: BEAST 1.10.5 XML file to perform travel history-aware discrete Bayesian phylogeographic analysis, on subtree B.1 of the full data set (see main text for the description of subtree B.1).

File Name: Supplementary Data 5
Description: mltree.iqtree2.tree: maximum-likelihood phylogenetic tree of the full data set in our study, consisting of the generated Rwandan genomes as well as those from its neighbouring countries, complemented with a representative set of genomes from the rest of the African continent as determined through an Africa-focused Nextstrain build. We used the COVID-19 release of IQ-TREE (v2.1.2) with automated model selection to estimate this unrooted phylogeny.
